# Analysis of the first test event in Santa Catarina, Brazil, in the context of the COVID-19 pandemic in July 2021: an experience report

**DOI:** 10.1590/s2237-96222022000200010

**Published:** 2022-08-01

**Authors:** Fabiana Schuelter-Trevisol, Graziela Liebel, Gabriella de Almeida Raschke Medeiros, Stella Maris Brum Lopes, Betine Pinto Moehlecke Iser, Eliane Traebert, Daisson José Trevisol, Jefferson Traebert, Eduardo Macário

**Affiliations:** 1 Universidade do Sul de Santa Catarina, Programa de Pós-Graduação em Ciências da Saúde, Tubarão, SC, Brazil; 2 Universidade do Vale do Itajaí, Programa de Pós-Graduação em Saúde e Gestão do Trabalho, Itajaí, SC, Brazil; 3 Secretaria de Estado da Saúde de Santa Catarina, Florianópolis, SC, Brazil

**Keywords:** COVID-19, Coronavirus Infections, Monitoring, Epidemiology

## Abstract

This study aimed to describe the management and results of the test event for evaluating relaxation of social distancing measures in Santa Catarina, Brazil. This is an experience report that described results of the test event carried out in July 2021 and for which the participants underwent real-time polymerase chain reaction diagnostic testing 72-48 hours before the event and had follow-up for 15 days afterwards. The outcomes analyzed were SARS-CoV-2 infection up to 14 days after the event and presence of symptoms. Among 313 participants, the mean age was 45.1 years and 54.3% were female. During follow-up, 7.7% (24) of the contacted participants reported symptoms compatible with infection, but of the 240 who attended post-event testing, none of the results detected the presence of the virus. No post-event COVID-19 cases were reported. We suggest that other test events be carried out to evaluate the recommendations indicated.

**Study contributions**

**Main results**

Although 7.7% of participants reported symptoms during the follow-up period, no COVID-19 cases were detected after the test event in Santa Catarina.

**Implications for services**

The evaluation of the safety protocol adopted in the event contributed to the resumption of activities with large audiences, indicating that the use of standardized masks and the vaccination strategy can guarantee the audience’s safety.

**Perspectives**

For as long as there is viral circulation, the holding of large events depends on the adequate immunization of the audience and the appropriate use of masks indoors.

## Introduction

In early 2020, the World Health Organization (WHO) declared novel coronavirus (SARS-CoV-2) infection to be a Public Health Emergency of International Concern, which was to become the COVID-19 pandemic.^1^

By the end of 2020, the first SARS-CoV-2 vaccines were provisionally registered for use in humans worldwide. In Brazil, their registration took place in early 2021.^2^ With the advance of vaccination coverage throughout 2021, the possibility arose of reviewing health protocols, with reduction of some restrictive measures and gradual resumption of economic activities.

Within the context of the return to cultural activities, the Santa Catarina State Government held the state’s first test event, at the Integrated Cultural Center (*Centro Integrado de Cultura - CIC*), at a concert held by the Camerata Florianópolis orchestra. On the day of the concert, July 29, 2021, according to the Santa Catarina Epidemiological Bulletin the state had already confirmed 1,112,629 COVID-19 cases, which corresponded to a rate of 155,291 cases per 1 million inhabitants. The total number of deaths at that time was 17,962, which represented a mortality rate of 2,507 deaths per 1 million inhabitants and a case fatality ratio of 1.61%. With regard to active cases, Santa Catarina presented a downward trend and 74.5% adult intensive care units (ICU) bed occupancy. By then, more than 4.9 million doses of vaccine had been administered in the state.^3^ The national vaccination strategy was conducted by age strata, in descending order, and on the date of the test event the population in their forties had priority, immediately after health professionals, people with comorbidities and other subgroups provided for in the National Immunization Plan.^4^

This was both a cultural and a scientific event, where a safety protocol was established to minimize the risk of COVID-19 transmission among the participants, and to monitor the results. The analysis of the regulations and the results of the protocol adopted during the concert was carried out by a working group of technicians and researchers from Santa Catarina, with the purpose of evaluating the effectiveness of individual and collective protection measures, as well as monitoring the occurrence of COVID-19 cases following the event.^5^

Based on the above, the objective of this experience report was to describe the management and results of the first test event aimed at relaxation of social distancing measures in the context of the COVID-19 pandemic, in the state of Santa Catarina, Brazil.

## Methods

This is an experience report of the first test event held in Santa Catarina in the context of the COVID-19 pandemic, on the occasion of the Camerata Florianópolis concert, held on July 29, 2021, at Teatro Ademir Rosa/CIC, in the city of Florianópolis, the state capital city. The duration of the event was 90 minutes, with maximum occupancy of the audience limited to 60% of the seats available in the auditorium.

All those involved gave their consent to participate by signing a free and informed consent form, before answering a questionnaire available through BlueTicket®, to be completed at the time of online registration for the event. The questionnaire requested individual information and vaccination history. Some of this information was not considered mandatory, such as age, brand of vaccine used, or the participant’s complete address.

The eligibility criteria for the concert spectators were: (i) being resident in the Greater Florianópolis region; (ii) presentation of their vaccination status, having been fully vaccinated for at least 21 days, validated by cross-checking the information held on the vaccination databases of the Santa Catarina State Health Department (SES/SC); (iii) undergoing a real-time polymerase chain reaction (RT-PCR) diagnostic test for detection of SARS-CoV-2 between 72 and 48 hours before the event and on the 4^th^ day after the event, as scheduled by the test organizers; and (iv) compliance with the health regulations in force, and signing the free and informed consent form. Eligibility criteria (iii) and (iv) were also applied to the support team and the musicians, but they were not required to meet eligibility criteria (i) and (ii). The use of facemasks (FFP2 with no valve), however, was compulsory for all involved during the entire event, and masks that did not guarantee complete coverage of the nose and mouth were not accepted. The support team had masks available for replacement if necessary. 

The list of those involved in the test event was sent in advance for labeling and control to the Central Public Health Laboratory (*Laboratório Central de Saúde Pública - LACEN*), linked to the SES/SC. The collection of material for serology was performed by the Universidade do Vale do Itajaí Clinical Analyses Laboratory School (*Laboratório Escola de Análises Clínicas*), with enough staff to perform ten tests simultaneously at the CIC. The analysis was performed by LACEN-SES/SC, both pre-event and on the 4^th^ day post-event.

Participants were excluded from the study if they had (i) a detectable test result or had signs or symptoms consistent with COVID-19 in the seven days prior to the event, (ii) body temperature higher than 37.4 ºC at the time of entering the event, or (iii) those who did not comply with the support team’s recommendations (no QR code at the event entrance; no identity document; no mask).

The space was organized with three independent entrances and route signs; at each entrance, there were three people ready to guide the public. The seats were arranged with interspersed seating, to ensure physical distancing. Showing the QR code at check-in enabled traceability in case of positive post-event test results. Consumption of food and drinks within the event building was not allowed in order to prevent facemasks from being removed. At the end of the event, the audience was instructed by the master of ceremonies on how to leave so as to avoid crowding.

Check-in via QR code was available for the support staff and musicians at circulation points in the theater, and registration on the platform was done with the Individual Taxpayer Registry (*Cadastro de Pessoas Físicas - CPF*) number, phone number and e-mail.

The follow-up period began following the event. Follow-up was carried out by the National Commercial Learning Service (*Serviço Nacional de Aprendizagem Comercial - SENAC*) team on day 3, 8 and 14 after the event. The follow-up period ended on the 15^th^ day after the event. Follow-up involved participants being contacted by a messaging app and asked to provide the following information: full name; CPF; city of residence; and whether they were experiencing any signs or symptoms related to COVID-19. Contact was always made at 8:30 p.m. and those contacted had until 12:00 a.m. the next day to respond.

After this period, the team analyzed the responses and made a list of people who had not answered the questionnaire, for further contact via phone calls. The state epidemiological surveillance service was notified of symptomatic cases for follow-up in the municipalities where they lived.

The study’s primary outcome of interest was SARS-CoV-2 infection at 14 days after the event, confirmed by RT-PCR testing, whereby the result was either “detectable” or “not detectable”. The secondary outcome was the reporting of symptom(s) related to the possibility of infection within 14 days after the concert, defined by reporting tiredness or headache or sore throat or shortness of breath or cough or fever or chills or anosmia or ageusia or diarrhea.

The other variables described were: sex (male; female); age (in completed years, then categorized into age groups: 19-39, 40-59, and ≥ 60 years); brand of anti-SARS-CoV-2 vaccine used (Coronavac®; Janssen®; Astrazeneca®; Pfizer®); date of the last dose received (or single dose, if applicable); and presence of at least one of the following symptoms, within 7 days before the event and 14 days post-event (no; yes), namely, tiredness, headache, sore throat, shortness of breath, cough, fever, chills, anosmia, ageusia and/or diarrhea.

All the data were input into a database, prepared using the Microsoft Office Excel application, and were later exported to SPSS version 18.0 (Armonk, New York, USA), for the purpose of descriptive analysis. Quantitative variables were described in measures of central tendency and dispersion, while qualitative variables were described according to frequency and percentage.

The study project was submitted to the Universidade do Vale do Itajaí Research Ethics Committee and approved as per Opinion No. 4.866.547 (Certificate of Submission for Ethical Appraisal No. 49977421.6.0000.0120), on July 26, 2021.

## Results

**Figure 1:**
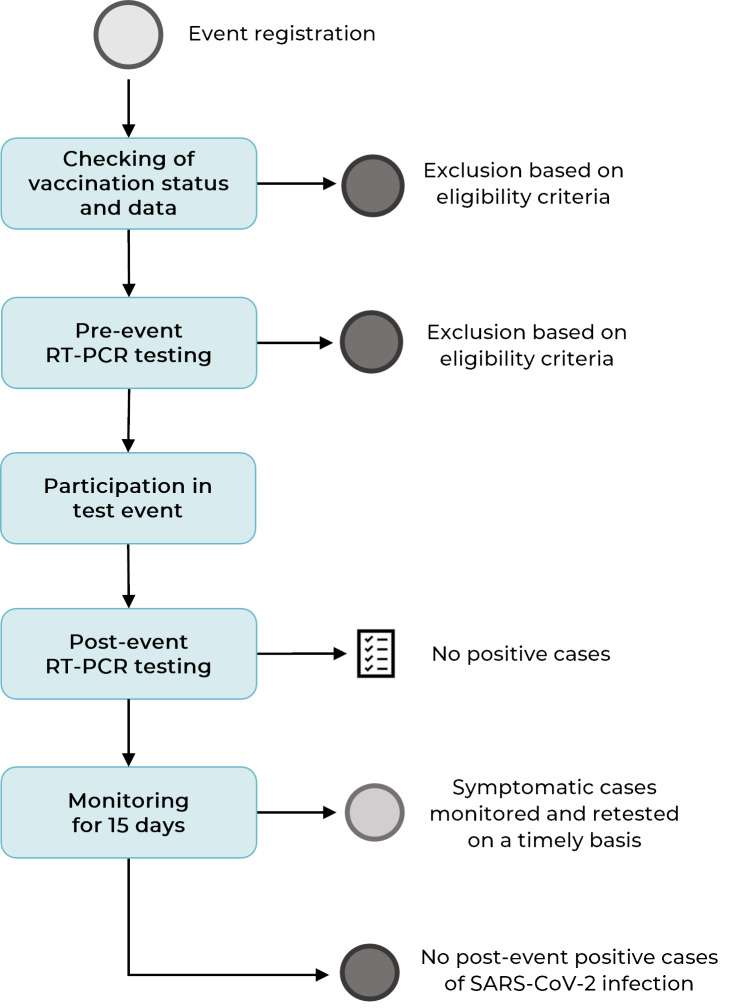
Flowchart showing selection of the participants of the first test event in the context of the COVID-19 pandemic, the Camerata Florianópolis concert, Santa Catarina, Brazil, July-August 2021

A total of 313 individuals participated in the study. Of the total enrolled, 54 were excluded for not meeting the eligibility criteria, especially if they had not been fully vaccinated for at least 21 days or if they did not reside in Greater Florianópolis. In the pre-event testing, one member of the support team had a detectable test result for SARS-CoV-2 infection, and two others had indeterminate test results, and all three were replaced by the organizers. There were no refusals to be tested. No musicians or guests had SARS-CoV-2 infection prior to the test event. Some people who registered did not attend the event, and there were no exclusions when entering the venue. Figure 1 shows the steps in which the sample was built.

Mean age among the participants was 45.1 years (standard deviation: ± 16), ranging from 19 to 86 years. However, information on age was not available for 69.0% of the participants. 

Table 1 shows participant distribution according to the following variables: ‘sex’, ‘age’, ‘vaccine brand used’ and event ‘participant category’, most of whom had registered voluntarily, were female and had been vaccinated with Coronavac®.

**Table 1:** Description of the participants (n = 313) of the first test event in the context of the COVID-19 pandemic, the Camerata Florianópolis concert, Santa Catarina, Brazil, July-August 2021

Variable	n	%
**Sex**		
Male	143	45.7
Female	170	54.3
**Age (in years)**		
19-39	77	24.6
40-59	91	29.1
≥ 60	48	15.3
Not informed	97	31.0
**Vaccine brand used**		
Coronavac®	126	40.3
Astrazeneca®	48	15.3
Pfizer®	7	2.2
Janssen®	49	15.7
Not vaccinated/not informed^a^	86	26.5
**Participant category**		
Audience	230	73.5
Support team	61	19.5
Musicians	22	7.0

a) Full vaccination was not obligatory for the support team or musicians.

On the 4^th^ day after the event, 240 (76.7%) of those involved attended for RT-PCR testing for SARS-CoV-2, and none of their results were detectable. During the follow-up period, there were reports of signs or symptoms consistent with COVID-19, but no cases were confirmed when tested in a timely manner. Table 2 shows the results of the post-event period: 6.1% of the total study participants had symptoms at the first follow-up (between 3 and 5 days); 7.3%, at the second follow-up (between 8 and 10 days); and 7.7%, at the third follow-up (between 14 and 15 days). After excluding individuals who had not been contacted, these proportions rose to 6.8%, 9.1% and 10.7% respectively, at the first, second and third follow-ups.

**Table 2:** Symptomatology reported during the monitoring periods following the first test event in the context of the COVID-19 pandemic, the Camerata Florianópolis concert, Santa Catarina, Brazil, July-August 2021

Symptomatology	n (%)		
	1^st^ follow-up 3-5 days	2^nd^ follow-up 8-10 days	3^rd^ follow-up 14-15 days
No symptoms	257 (82.1)	229 (73.2)	201 (64.2)
Symptoms	19 (6.1)	23 (7.3)	24 (7.7)
Not contacted	37 (11.8)	61 (19.5)	88 (28.1)

The symptoms reported by the participants contacted during the follow-up periods are shown in Table 3. The most frequent symptom was nasal obstruction or rhinorrhea (56; 40.9%). There were variations between the three follow-up periods, whereby symptomatic people stopped presenting symptoms and vice versa. Of the total, 14 participants reported more than one sign or symptom.

**Table 3:** Distribution of symptoms reported by the participants of the first test event in the context of the COVID-19 pandemic, the Camerata Florianópolis concert, contacted in the post-event monitoring period, Santa Catarina, Brazil, July-August 2021

Symptoms^a^	n	%
Nasal obstruction or rhinorrhea	56	40.9
Headache	33	24.1
Sore throat	23	16.8
Cough	14	10.2
Tiredness	7	5.1
Diarrhea	4	2.9

a) More than one symptom may have been reported by the same participant.

## Discussion

In the first test event in Santa Catarina, no detectable cases of COVID-19 were found after the event. However, around 7.7% of individuals reported symptoms during the follow-up period. Analysis of these initial results requires caution, since this was an event carried out in a controlled environment and with a population that had been immunized beforehand.

It should be noted that on the date of the concert, July 29, 2021, the pandemic had low transmissibility in Santa Catarina, and there was no confirmation of the Delta variant in Brazil.^3^ New variants may require vaccine booster doses in order to maintain high levels of neutralizing antibodies.^6^ ^,^ ^7^ In countries where crowded events have been held, there have been peaks in transmission, most likely due to the lack of strict protocols being adopted or not being controlled by the organizers.^8^ ^,^ ^9^ In countries such as the United States, China and several European countries, where non-pharmacological measures (such as the use of masks in schools and open environments) have been abandoned, this has been followed by an increase in SARS-CoV-2 transmissibility, with new peaks in hospitalization and deaths.^10^ In the United States, Spain and the United Kingdom, vaccination has slowed down due to fear and resistance of many people to the use of immunoprophylaxis.^11^ ^,^ ^12^

The scarcity of other reports similar to this one in the scientific literature makes it difficult to compare the results found. Test events carried out in Brazil and internationally generally did not include follow-up and analysis of results, unlike the test event in Santa Catarina. However, we did find rules with similar characteristics in test events in the state of Ceará, where there was commitment to inspecting the event and monitoring participants for 14 days after the event, proof of full vaccination, measures to control access to the event at the entrance, distancing of 1.0 m between people and mandatory use of facemasks.^13^ Staff involved in holding parties and events, or working in hotels, bars and restaurants were also required to attend health protocol biosafety courses.^13^

It should be noted that although there were no recorded cases of post-event COVID-19, protocol deviations were noted in the way the event was conducted and also post-event that contributed to the limitations of the present study, as presented below.

Despite participants initially agreeing to post-event testing and follow-up by signing the free and informed consent form, not all of them attended post-event testing and/or did not respond to follow-up attempts. As such, the data presented refer to the proportion of participants who showed up for post-event testing and/or replied to the monitoring team. Therefore, the research team cannot guarantee that subsequent cases did not occur and/or were not registered, despite the tracing efforts made by the municipality’s health teams during the follow-up period. The short deadline for preparing the research project and the complexity of all the processes, involving many actors, may have generated information with some degree of inconsistency, which could represent selection or measurement bias. In this sense, the lack of more complete sociodemographic characterization and a 76.2% response rate for post-event testing stand out. These aspects require attention when planning other events of a similar nature.

The test event held in Santa Catarina was unprecedented in Brazil and covered a wide range of intentions. Through the event it was possible to evaluate a proposed safety protocol for the resumption of some activities with large audiences, using non-pharmacological measures such as the use of standardized masks, physical distancing, adequate ventilation and hygiene, in addition to the vaccination strategy itself, since being fully vaccinated was required as a prerequisite for participation in the event.^10^ ^,^ ^14^ The test event was planned in an interdisciplinary manner, with the participation of different sectors of government, civil society, services linked to economic activities, commerce, tourism, and events, along with universities and the health sector, in order to obtain a comprehensive evaluation and provide answers to questions that were still pressing. This was the first step towards the application of a protocol to be adopted at other times and in different scenarios, informing specific actions.

For these reasons, and based on our experience, we recommend the following for similar events:

- The entire eligible audience must prove that it is fully vaccinated.
- FFP2 masks without valves, N95 respirators or surgical masks must be used as they are recognized as being safer.^14^ ^,^ ^15^ As such, an efficient indoor air exchange system is necessary. Holding events in an open environment with good natural ventilation is preferable to holding them in closed venues. In 2022, use of masks in open and closed environments has become optional,^16^ although we must emphasize the importance of their use in large crowds and by individuals who are more vulnerable to COVID-19 - the elderly, immunosuppressed people and those with multiple comorbidities.
- Sanitizing utensils that may be shared, such as microphones, with sanitizers with a 70% concentration of ethanol.
- Ensuring physical distancing, with interspersed and properly signaled seats, respecting a minimum distance of 1.0 m between people in all environments, by reducing the capacity of the venue in order to meet this recommendation and avoid crowding; and ensuring a minimum distance of 2.0 m between musicians/artists and the audience.
- Consumption of food and drinks indoors must not be allowed, so as to prevent the audience from removing their masks.
- The existence of a team prepared to provide guidance on health regulations and enforce them is fundamental.
- If post-event traceability is considered, good Wi-Fi access must be ensured at the event location, as well as adequate connectivity guidelines for effective traceability.^17^

We conclude that the results of the first test event in the state of Santa Catarina should be interpreted with caution. Other test events, endowed with the necessary scientific rigor and greater control of data, can help to ensure the suggested recommendations. Finally, these recommendations are conditioned to the epidemiological moment of the COVID-19 pandemic.
